# Synergistic Effect between Cisplatin and Sunitinib Malate on Human Urinary Bladder-Cancer Cell Lines

**DOI:** 10.1155/2013/791406

**Published:** 2013-11-28

**Authors:** Regina Arantes-Rodrigues, Rosário Pinto-Leite, Lio Fidalgo-Gonçalves, Carlos Palmeira, Lúcio Santos, Aura Colaço, Paula Oliveira

**Affiliations:** ^1^Centre for the Research and Technology of Agro-Environmental and Biological Sciences (CITAB), University of Trás-os-Montes and Alto Douro, Vila Real, 5001-801 Vila Real, Portugal; ^2^Genetic Service, Cytogenetic Laboratory, Hospital Center of Trás-os-Montes and Alto Douro, 5000-508 Vila Real, Portugal; ^3^Department of Engineering, CMUTAD, University of Trás-os-Montes and Alto Douro, 5001-801 Vila Real, Portugal; ^4^Experimental Pathology and Therapeutics Group, Portuguese Institute of Oncology, 4200-072 Porto, Portugal; ^5^Health School, University Fernando Pessoa, 4249-004 Porto, Portugal; ^6^Department of Veterinary Sciences, CECAV, University of Trás-os-Montes and Alto Douro, 5001-801 Vila Real, Portugal

## Abstract

The aim of this paper is to analyse sunitinib malate *in vitro* ability to enhance cisplatin cytotoxicity in T24, 5637, and HT1376 human urinary bladder-cancer cell lines. Cells were treated with cisplatin (3, 6, 13, and 18 **μ**M) and sunitinib malate (1, 2, 4, 6, and 20 **μ**M), either in isolation or combined, over the course of 72 hours. 3-(4,5-Dimethylthiazol-2-yl)-2,5-diphenyl tetrazolium bromide assay, acridine orange, and monodansylcadaverine staining and flow cytometry were performed. The combination index (CI) was calculated based on the Chou and Talalay method. In isolation, cisplatin and sunitinib malate statistically (*P* < 0.05) decrease cell viability in all cell lines in a dose-dependent manner, with the presence of autophagic vacuoles. A cell cycle arrest in early S-phase and in G_0_/G_1_-phase was also found after exposure to cisplatin and sunitinib malate, in isolation, respectively. Treatment of urinary bladder-cancer cells with a combination of cisplatin and sunitinib malate showed a synergistic effect (CI < 1). Autophagy and apoptosis studies showed a greater incidence when the combined treatment was put into use. This hints at the possibility of a new combined therapeutic approach. If confirmed *in vivo*, this conjugation may provide a means of new perspectives in muscle-invasive urinary bladder cancer treatment.

## 1. Introduction

Urinary bladder cancer is a common malignancy of the urinary tract, being four times higher in men than in women [1]. Remarkable differences can be found in its incidence worldwide, while being predominately higher in developed countries such as North America and Western and Southern Europe [2]. At diagnosis, approximately 70% are nonmuscle invasive tumors [3], while the remaining 30% are muscle invasive and of these tumors about 10% of cases has a tendency to metastasize, with a poor prognosis [4]. The standard approach for muscle-invasive urinary bladder cancer treatment is based on a radical cystectomy with bilateral pelvic lymph node dissection. However, this treatment only offers 5-year survival in about 68% of patients [5]. Cisplatin-based chemotherapy is widely used. Gemcitabine plus cisplatin exert comparable activity and a lower toxicity profile when compared to the methotrexate, vinblastine, doxorubicin, and cisplatin (MVAC) regimen [6]. However, chemotherapy courses continue to produce unsatisfactory rates of recurrence and death. Thus, the simultaneously application of cisplatin with other anticancer drugs that target new signalling pathways has been investigated [7].

Sunitinib malate is an orally bioavailable molecule with the ability to block the intracellular tyrosine kinase domain of tyrosine kinase receptors. It is also responsible for inhibition of vascular endothelial growth factors receptors, plateletderived growth factor receptors, and stem cell factor receptor [8, 9]. Its therapeutic effects on urinary bladder cancer have already been assessed in two clinical studies of phase II cancers and showed clinical benefits [10, 11]. Nonetheless, there is a scarce of data available about the combination of sunitinib malate and cisplatin on urinary bladder cancer.

This investigation aims to analyse the *in vitro* effects of cisplatin and sunitinib malate in isolation and in combination, on one human nonmuscle invasive urinary bladder-cancer cell line (5637) and on two human muscle-invasive urinary bladder-cancer cell lines (T24 and HT1376).

## 2. Materials and Methods

### 2.1. Urinary Bladder-Cancer Cell Lines and Culture Conditions

The study was performed on the 5637, T24, and HT1376 urinary bladder-cancer cell lines. T24 cell line was provided by DSMZ, Düsseldorf, Germany; 5637 and HT1376 cell lines were kindly provided by Dr. Paula Videira of the Universidade Nova de Lisboa, Lisboa, Portugal. Monolayer cultures of the three cell lines were maintained in RPMI 1640 medium (PAA, Pasching, Austria), supplemented with 10% heat inactivated fetal bovine serum (Biological Industries, Kibbutz Beit Haemek, Israel), 100 *μ*g/mL streptomycin (Biological Industries), 100 U/mL penicillin (Biological Industries), and 2 mM L-Glutamine (Sigma Aldrich, St. Louis, MO, USA). The cultures were maintained in a humidified 5% CO_2_ incubator at 37°C.

### 2.2. Cisplatin and Sunitinib Malate Exposure

T24, 5637, and HT1376 urinary bladder-cancer cells were exposed to 3, 6, 13, and 18 *μ*M cisplatin (Teva Pharma, Portugal) and 1, 2, 4, 6, and 20 *μ*M sunitinib malate (Sigma Aldrich, St. Louis, Mo, USA), in isolation and over the course of 72 hours to assess dose-response profiles. For the cell viability combined assay, 3, 6, and 13 *μ*M cisplatin was used with each concentration of sunitinib malate (1, 2, and 4 *μ*M). For morphological analysis, acridine orange, and monodansylcadaverine (MDC) staining, the lowest concentration of cisplatin (3 *μ*M) was used simultaneously with 1 *μ*M of sunitinib malate. For flow cytometry assay, 3 *μ*M cisplatin was combined with 1, 2, and 4 *μ*M of sunitinib malate.

Cells growing in the complete medium alone were processed in the same way as the treated cells, in the case of all the methodologies, and cytotoxic effects were analyzed immediately after drug exposure was ceased.

### 2.3. Morphological Analysis

Urinary bladder-cancer cell lines (2 × 10^4^ cells/mL) were cultured in the presence of the lowest concentrations of cisplatin and sunitinib malate, in isolation or combined. A light inverted microscope (Axiovert 25, Carl Zeiss, Germany) was used to observe the cells in culture, in order to detect cells confluence and differences in their appearance.

### 2.4. 3-(4,5-Dimethylthiazol-2-yl)-2,5-diphenyl Tetrazolium Bromide (MTT) Assay

The effect of both drugs was evaluated based on the MTT assay. Cells were seeded into each well of a 96-well flat-bottom microtiter (Sarstedt, Newton, NC, USA) at a density of 2 × 10^4^ cells/mL and allowed to adhere overnight. Cells were treated with cisplatin (3, 6, 13, and 18 *μ*M) and sunitinib malate (1, 2, 4, 6, and 20 *μ*M), in isolation or combined. At the end of the treatment, the MTT (Sigma Aldrich, St. Louis, USA) dye working solution (10 *μ*L/well at 5 mg/mL) was added to each well and plates were incubated for 4 additional hours. The medium was removed and the formazan crystals generated were solubilized by adding 100 *μ*L/well of dimethylsulfoxide (Sigma Aldrich) for 5 minutes. Absorbance values at 492 nm were determined using an automatic ELISA plate reader (Multiskan EX, Labsystems). The percentage of cell viability was calculated as (absorbance of treated cells/absorbance of untreated cells) ×100.

### 2.5. Drug Combination Studies

For the study of synergism between cisplatin and sunitinib malate on cell growth inhibition of T24, 5637, and HT1376 cells, a combination index (CI) was performed using the data obtained from MTT assay. Drug combination studies were based on concentration-effect curves generated as a plot of the fraction of unaffected cells versus drug concentration, in accordance to the Chou and Talalay (1984) method [12], using the following CI equation: CI = (D)_1_/(Dx)_1_ + (D)_2_/(Dx)_2_ + (D)_1_(D)_2_/(Dx)_1_(Dx)_2_, where (D)_1_ and (D)_2_ are the concentrations of sunitinib malate and meloxicam that exhibit a determined effect when applied simultaneously to the cells and (Dx)_1_ and (Dx)_2_ are the concentrations of the same drugs that exhibit the same determined effect when used in isolation. The CI values indicate a synergistic effect when <1, an antagonistic effect when >1, and an additive effect when equal to 1.

### 2.6. Acridine Orange Staining

Acridine orange is a fluorescent dye which stains cytoplasm and nucleus by bright green, while acidic compartments (such as lysosomes and autolysosomes) stain bright red. Cells (2 × 10^4^ cells/mL) were seeded on sterilized glass coverslip (8 mm), cultured for 24 hours, and treated with drugs, in isolation or combined, for 72 hours. The medium was removed and acridine orange (Sigma, Karlsruhe, Germany) was added to the cells at 1 *μ*g/mL, at 37°C and for 10 minutes. Subsequently, cells were washed with phosphate-buffered saline (PBS) and immediately analysed using a fluorescence microscope (Nikon Eclipse E400, Tokyo, Japan).

### 2.7. Monodansylcadaverine (MDC) Staining

Autophagy induced by cisplatin and sunitinib malate, in isolation or combined, was observed with the autofluorescent substance MDC (Sigma, Karlsruhe, Germany). MDC moves freely to cross-biological membranes and accumulates in autophagic vacuoles [13]. Cells (2 × 10^4^ cells/mL) were seeded in sterile coverslips (8 mm), incubated for 24 hours, and then treated with the both drugs, as single agents or in combination, for 72 hours. Autophagic vacuoles were labelled with MDC by incubating cells with 25 *μ*M MDC for 1 hour at 37°C. The cells were washed three times with PBS and immediately analysed using a fluorescence microscope (Nikon Eclipse E400, Tokyo, Japan).

### 2.8. Flow-Cytometry Analysis

Cells (1 × 10^6^ cells/mL) were seeded in 6-well plates and allowed to adhere overnight. Subsequently, the medium was removed and 3 *μ*M cisplatin, in isolation or in combination with sunitinib malate (1, 2, and 4 *μ*M) was applied. Cell-cycle distribution and apoptosis were analyzed by flow cytometry as previously reported [14]. DNA-content histograms were analysed with CXP software (Beckman Coulter). Cells with a lower DNA content than that of G_0_/G_1_ phase cells were considered to be apoptotic (sub-G_0_/G_1_) [15]. Each independent experiment was performed in triplicate.

### 2.9. Statistical Methods

Statistical analysis was carried out using the SPSS 17.0 statistical software (SPSS Inc., USA). The equality of variances was tested by the Levene *F* test and the statistical significance of differences between the treatment and control groups was determined by Dunnett's multiple comparison post hoc test for the MTT assay. The Pearson product-moment correlation coefficient was used to evaluate the correlation (linear dependence) of the cell-cycle and drugs concentration. Data obtained from MTT assay and used to evaluate the interaction between cisplatin and sunitinib malate were analyzed using the MATLAB software (version 7.9, R2009b). Statistical significance was set at *P* < 0.05.

## 3. Results

### 3.1. Morphological Alterations

**Figure 1:**
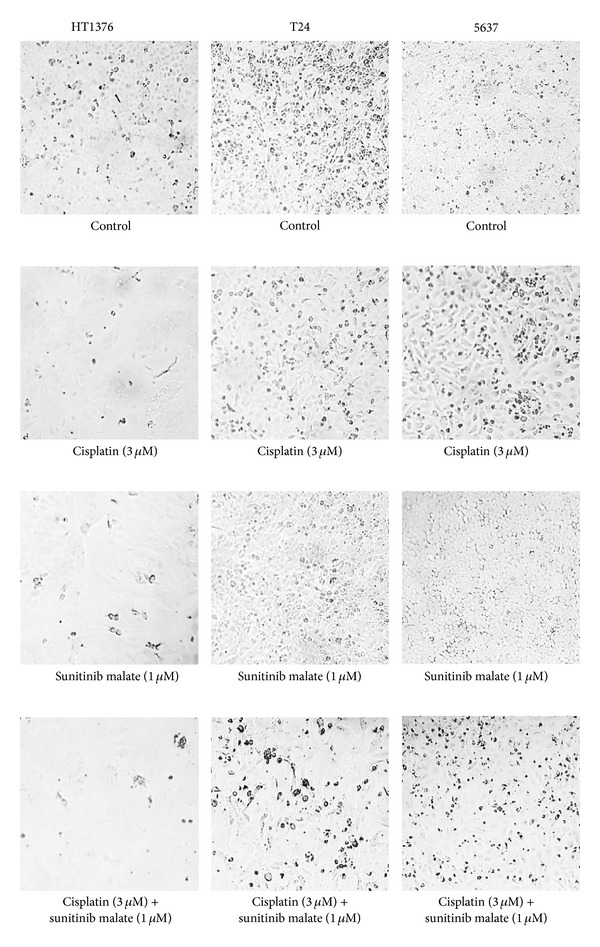
HT1376, T24, and 5637 urinary bladder-cancer cell lines culture, in the absence (control) or in the presence of cisplatin and sunitinib malate, in isolation or combined, under a light inverted microscope. Original magnification 10x.

Cisplatin and sunitinib malate, in isolation, induced a decrease cell population when compared to untreated cells. In combined treatment, a slight decrease of cell confluence with an increase number of granulated cells was observed, when compared with the other culture flasks with isolated drugs. These features were more visible on T24 and 5637 cell lines. The surface in control flask was confluent with visible undergoing division cells (Figure 1).

### 3.2. Isolated Effects of Cisplatin and Sunitinib Malate on Urinary Bladder-Cancer Cell Viability

T24, 5637, and HT1376 cell lines in the exponential growth phases were exposed to different concentrations of cisplatin and sunitinib malate, in isolation or combined, and the effect on cell viability was examined after 72 hours of culture.

**Figure 2:**
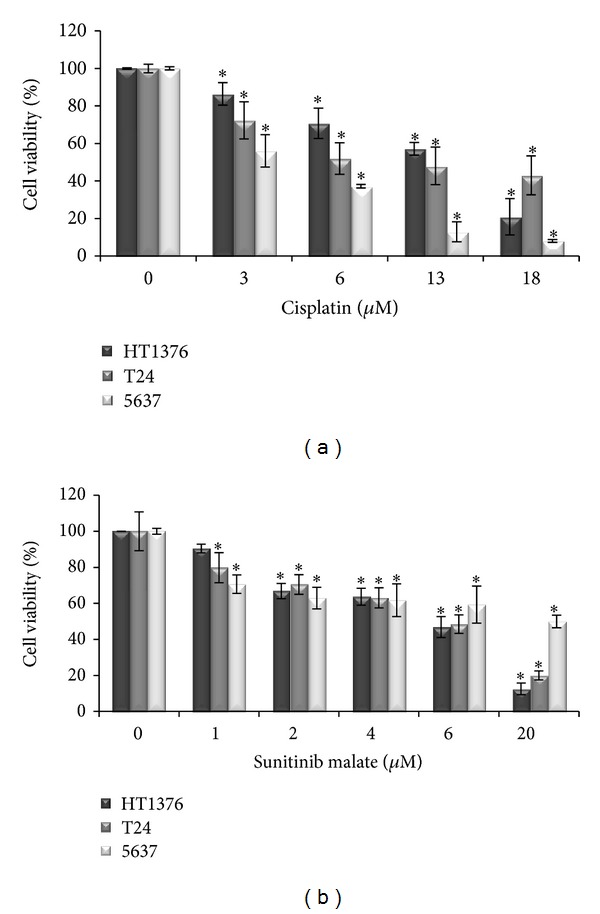
Isolated effects of cisplatin (a) and sunitinib malate (b) on urinary bladder-cancer cell lines viability, assessed by using the MTT assay. The data shown and bars represent the mean values ± SD (SD: standard deviation). *P < 0.05 versus untreated cells.

Cisplatin decreased cell viability in all the three cell lines in a dose-dependent manner. The 5637 cell line was the most sensitive, with only 8% of cell viability at the highest concentration tested (18 *μ*M). In the three cell lines, statistically significances (*P* < 0.05) were found in all the concentrations tested when compared with untreated cells (Figure 2(a)).

Sunitinib malate induced a concentration-dependent inhibitory effect on cell viability, with a very similar pattern response between the three cell lines. However, the 5637 cell line was the most resistant at the higher concentration applied (20 *μ*M). In all the cell lines, statistically significant values were found when compared with untreated cells (*P* < 0.05), with the exception at the lowest concentration in the HT1376 cell line (*P* = 0.171) (Figure 2(b)).

### 3.3. Combined Effects of Cisplatin and Sunitinib Malate on Urinary Bladder-Cancer Cell Viability

**Figure 3:**
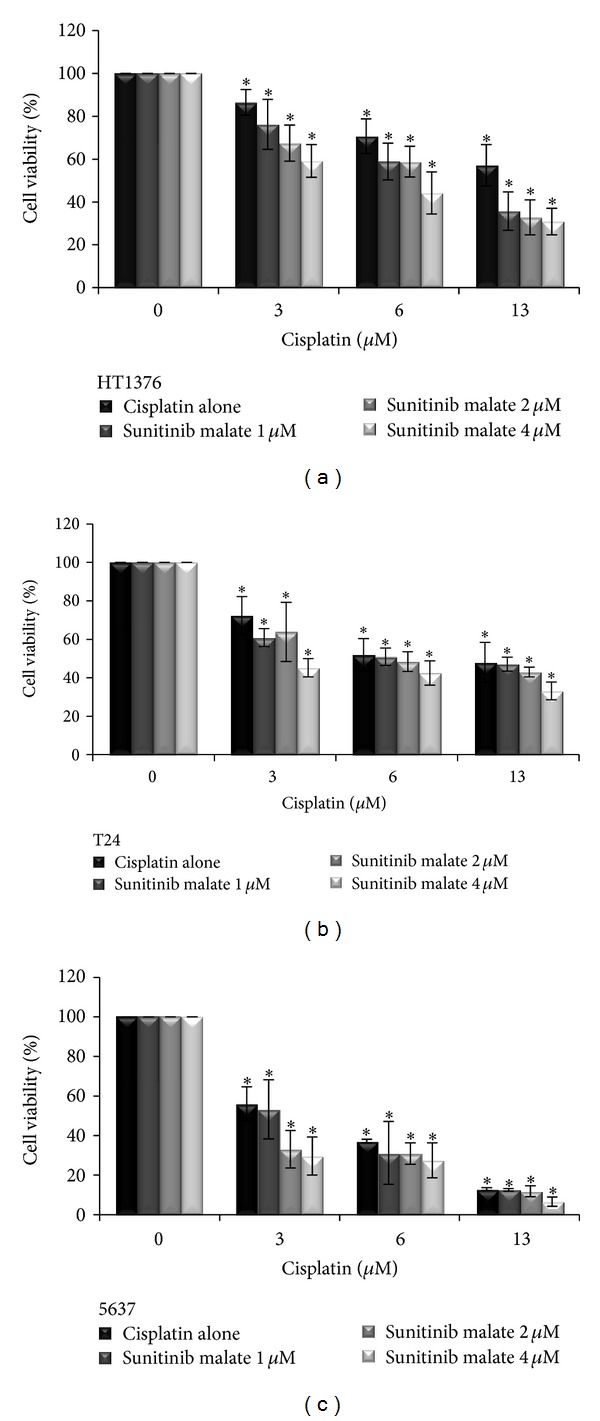
Combined effects of cisplatin (3, 6, and 13 *μ*M) and sunitinib malate (1, 2, and 4 *μ*M) on HT1376, T24, and 5637 urinary bladder-cancer cell lines viability, assessed by using the MTT assay. The data shown and bars represent the mean values ± SD (SD: standard deviation). *P < 0.05 versus untreated cells.

The simultaneous treatment of urinary bladder-cancer cells to cisplatin (3, 6, and 13 *μ*M) and sunitinib malate (1, 2, and 4 *μ*M) decreased the cell viability rate in the three cell lines when compared with each drug in isolation (Figure 3). The 5637 cell line was the most sensitive to drugs used in association, even at the lower cisplatin concentration tested (3 *μ*M) with 1, 2, and 4 *μ*M concentration of sunitinib malate. The 5637 survival rates for this combination were averaged as 53.2%, 33.1%, and 29.7%, followed by the T24 (60.9%, 63.9%, and 45.2%) and HT1376 (76.2%, 67.4%, and 59.1%) cell lines, respectively. All the combinations are statistically significant when compared with the control group (*P* < 0.05).

### 3.4. Combination Index

In order to analyse the type of interaction (synergic, additive, or antagonistic) between the cisplatin and sunitinib malate in combination at 72 hours on T24, 5637, and HT1376 cell lines, we implemented on MATLAB the method developed by Chou and Talalay (1984) [12]. The CI_50_ values computed for HT1376, T24, and 5637 cell lines were 0.96, 0.96, and 0.89, respectively (Table 1). Therefore, the combined use of cisplatin and sunitinib malate was synergistic on the growth inhibition of the three cell lines. Dose reduction index_50_ (DRI_50_) represents the magnitude of dose reduction obtained for the 50% growth inhibitory effect in combination setting as compared to each drug alone. In our experiments, DRI_50_ of cisplatin and sunitinib malate were equal to 5.51 and 1.5 in HT1376, 2.86, and 2.19 in T24, and 1.25 and 20 in 5637 cells, respectively, when the two drugs were used in combination (Table 1). These results demonstrate that a synergistic interaction can be verified on cell viability when the two drugs are used in a concomitant schedule.

### 3.5. Detection of Autophagy by Acridine Orange Staining

**Figure 4:**
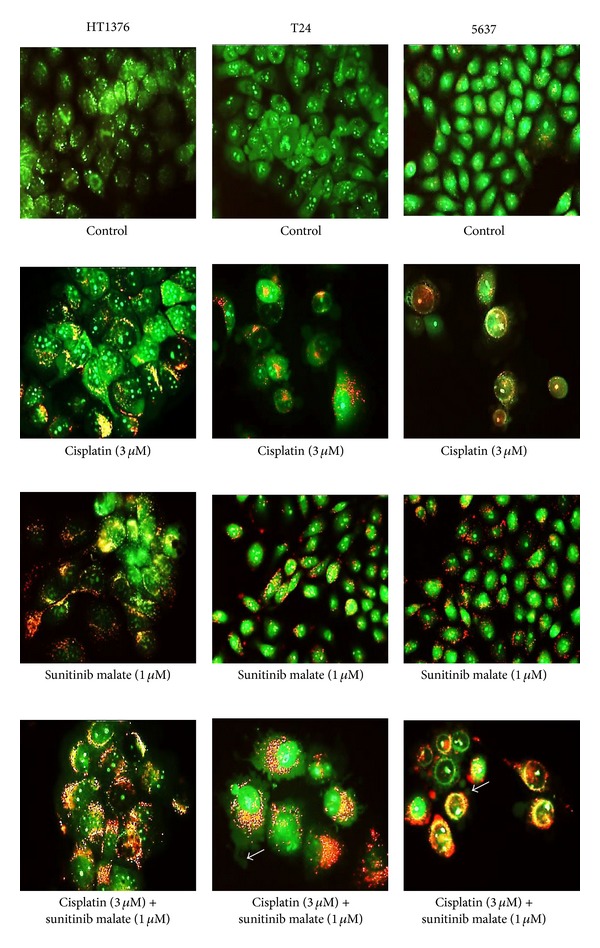
Fluorescence images obtained from cells exposed to cisplatin and sunitinib malate (green: cytoplasm and nucleus cells; red: acidic compartments; white arrow: membrane blebbing). Original magnification 400x.

In untreated cells acidic vesicular organelles were not observed. In isolation, the acidic vesicular organelles were observed in the three cell lines, after incubation with cisplatin (3 *μ*M) and sunitinib malate (1 *μ*M). However, this effect was more evident on sunitinib malate treated cells. Upon exposure to combined treatment, increased acidic vesicular organelles was detected in the three cell lines. Moreover, with this staining it was possible to observe that cells exposed to cisplatin, in isolation, showed the formation of membrane blebbing, which are morphological alterations consistent with apoptosis. This detection had a higher incidence on T24 and 5637 cell lines and markedly increased in the combined treatment (Figure 4).

### 3.6. Detection of Autophagy by MDC Staining

**Figure 5:**
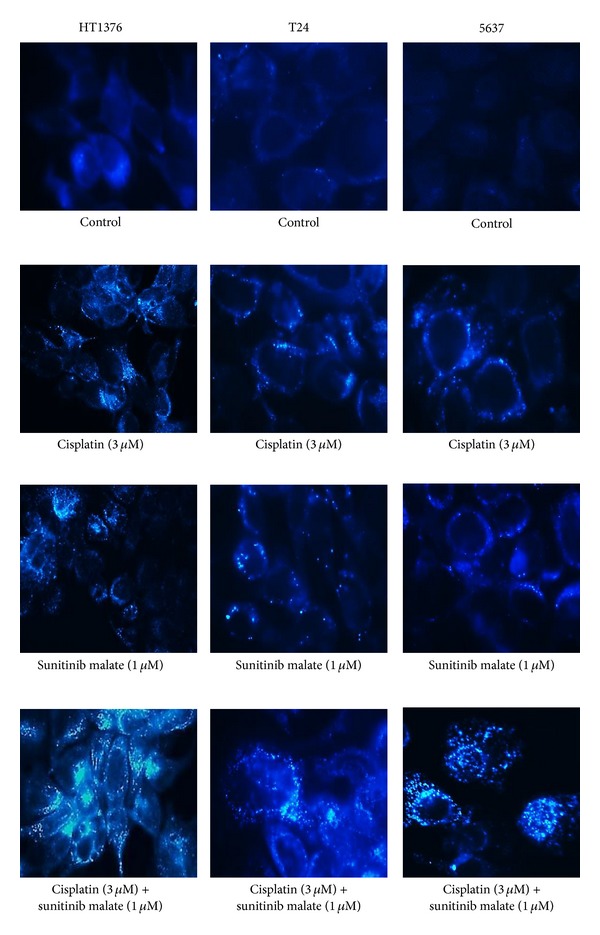
Fluorescence images obtained from cells exposed to cisplatin and sunitinib malate. Autophagosomes are pictured as distinct dot-like fluorescent structures (DAPI). Original magnification 400x.

MDC staining allows us to visualize the mature autophagic vacuoles. In untreated cells, autophagic vacuoles were inexistent. MDC-labelled vacuoles were detected after 72 hours of treatment with cisplatin and sunitinib malate, in isolation, in the three urinary bladder-cancer cell lines. In the simultaneous treatment, an increased number of MDC-labelled vesicles (fluorescent particles) was observed in cytoplasm and perinuclear regions (Figure 5).

### 3.7. Cell-Cycle Distribution and Sub-G_0_/G_1_-Fraction

The pattern of cell distribution through the several phases of the cell cycle was different in the three cell lines depending on the drug treatment applied (Table 2). Untreated cells were predominantly at G_0_/G_1_-phase, with 75.9%, 91.6%, and 81.1%, on HT1376, T24, and 5637 cell lines, respectively. With 3 *μ*M cisplatin the percentage of cells in G_0_/G_1_-phase decreased to 45.9% (HT1376), 71.5% (T24), and 24.1% (5637). This decrease was accompanied by an early S-phase and sub-G_0_/G_1_ arrest, particularly for 5637 and T24 cell lines. Regarding sunitinib malate treatment, two cell cycle effects were observed for all the cell lines. First the cells in interphase were mainly in G_0_/G_1_-phase (Table 2) and second an increase in sub-G_0_/G_1_-fraction was observed.

The effect of the drug combination is also concentration dependent as shown by the increase sub-G_0_/G_1_-fraction, which is considered to be a marker of apoptotic cell death. On HT1376 and T24 cell lines, a positive correlation was found between combined treatment and the increase of cells in the sub-G_0_/G_1_-fraction, with statistically significant results ((*r* = 0.947; *P* = 0.015) and (*r* = 0.959; *P* = 0.010)), respectively, (Table 3).

## 4. Discussion

Urinary bladder cancer is a common malignancy and remains a challenge despite significant therapeutic advances [16]. Thus, novel targeted therapies are sorely required to further improve the effectiveness of urinary bladder cancer chemotherapy. Preclinical models play a crucial role in this setting [17] and urinary bladder-cancer cell lines have been invaluable research tools to evaluate the efficacy of new drugs [18–20]. In the present study, we investigated if sunitinib malate could strengthen cisplatin cytotoxicity, using as *in vitro* models three human urinary bladder-cancer cell lines representative of human urinary bladder tumors: one nonmuscle invasive cell line (5637) and two muscle-invasive cell lines (T24 and HT1376).

The treatment of HT1376, T24, and 5637 urinary bladder-cancer cell lines with cisplatin and sunitinib malate, in isolation, significantly (*P* < 0.05) reduced cell viability in a dose-dependent manner as already reported in our previous studies [7, 20]. Similar results were described on 5637, J82, HT1197, and 253J urinary bladder-cancer cell lines [21], as well as on A2780 and OVCAR3 ovarian cancer cells [22] when exposed to cisplatin in isolation. Concerning sunitinib malate, its effect was already reported on 5637 [23], TCC-SUP, HTB5, HTB9, T24, UMUC14, SW1710, and J82 urinary bladder-cancer cell lines [24]. Comparable results were reported for other neoplastic cells, such as medullary and papillary thyroid [25], pancreatic adenocarcinoma [26], and non-small-lung cancer cell lines [27]. Also in *in vivo* studies, sunitinib malate was effective in mouse with small cell lung cancer [28] and in a mouse orthotopic urinary bladder tumor model [29].

We further investigated if the cytotoxic activity of cisplatin and sunitinib malate is mediated by autophagy and apoptosis. A rapid approach to testing whether autophagy may be occurring is to measure cellular acidification by using acridine orange and MDC staining. Both methods revealed an increased presence of acidic vesicles organelles and autophagosomes in cells treated with both drugs in isolation, when compared with untreated cells. This suggests that cisplatin and sunitinib malate may exert its effects through autophagy. In fact, the autophagic effect induced by sunitinib malate alone was previously reported on muscle cardiac cell lines [30]. Associated with autophagy induction is cell accumulation in G_0_/G_1_-phase of the cell cycle [31, 32]. Flow cytometry was used to evaluate the cell cycle kinetics and we detected a cell cycle arrest in early S-phase and in G_0_/G_1_-phase, in exposed cells to cisplatin and sunitinib malate, respectively. Concerning the isolated cisplatin treatment, our finding is consistent with previously published results on HeLa cells [33]. However, a cell cycle arrest in G_2_-phase phase was described in breast, testicular, head, and neck cell lines [34, 35]. In our study, the treatment of cells with sunitinib malate leads to an accumulation of cells in G_0_/G_1_-phase, an effect that was reported on A549 human non-small-cell lung cancer cells [27]. Moreover, for both drugs in isolation, we obtained an increase percentage of cells in sub-G_0_/G_1_-fraction in the three cell lines, suggesting that both agents induce apoptosis. This apoptotic effect was already reported on urinary bladder [7, 36, 37] and ovarian cancer cells [38] after exposure to cisplatin. Apoptosis induced by sunitinib malate was previously reported on 5637 [23] and T24 [39], on serous papillary epithelial ovarian cells [38], and on nasopharyngeal cancer cell lines [40].

Concerning the simultaneous treatment of both drugs, to our best knowledge, there are no data available on muscle-invasive urinary bladder-cancer cell lines (HT1376 and T24). Sonpavde and collaborators (2009) [23] have already tested this approach on the 5637 nonmuscle invasive urinary bladder-cancer cell line, with encouraging results. We also used this cell line in our study and furthermore we tested on to muscle-invasive cell lines and analyze the synergistic effect based on Chou and Talalay method [12]. A synergistic interaction (CI < 1) with a combined schedule of cisplatin and sunitinib malate was obtained in the three cell lines, more pronounced in the 5637 cell line, with lower cell viability when compared with each drug in isolation, as demonstrated by MTT assay. This synergistic effect can be explained by the similar effects of both drugs, as verified by the cell cycle arrest in the G_0_/G_1_ and early S-phase, after treatments. Furthermore, the sub-G_0_/G_1_-fraction of the three cell lines showed a higher apoptotic index, which is consistent with a concomitant increase of autophagy observed by the acridine orange and the MDC staining. This conjugation of cisplatin and sunitinib malate was described in gastric cells, with also beneficial effect [41].

The different response obtained on the three cell lines, being the nonmuscle invasive urinary bladder-cancer cell line the most sensitive, may be explained by the different origin of the cell lines. Nevertheless, the two muscle-invasive urinary bladder-cancer cell lines presented a similar and improved pattern response, when compared with each drug in isolation.

In conclusion, and although this is a preliminary study, this is the first report that provides valid results concerning the combination of cisplatin and sunitinib malate, on muscle-invasive urinary bladder-cancer cell lines. This synergistic interaction leads to a reduced cell viability, increased autophagy, and apoptosis. Future molecular *in vitro* and *in vivo* studies are required to confirm these results. The present data opens a new possible approach in the treatment of muscle-invasive urinary bladder cancer.

## Figures and Tables

**Table 1:** Combination index^a^ (CI) and dose reduction index (DRI) values for cisplatin and sunitinib malate combination.

Cell lines	Cisplatin (IC_50_ *μ*M)	Sunitinib malate (IC_50_ *μ*M)	CI_50_	DRI_50_	Interpretation
HT1376	18.2	6	0.96	Cisplatin: 5.51 Sunitinib malate: 1.5	Synergism
T24	9.44	6	0.96	Cisplatin: 2.86 Sunitinib malate: 2.19	Synergism
5637	4.12	20	0.89	Cisplatin: 1.25 Sunitinib malate: 20	Synergism

^a^CI_50_ is a combination index for 50% effect, used for quantifying synergism, additivity, and antagonism.

**Table 2:** Cell-cycle distribution of HT1376, T24, and 5637 urinary bladder-cancer cell lines, after treatment with cisplatin and sunitinib malate, in isolation or combined. G_0_/G_1_, S, and G_2_/M values are mean ± SD of the three independent experiments.

	HT1376			T24			5637		
	G_0_/G_1_	S	G_2_/M	G_0_/G_1_	S	G_2_/M	G_0_/G_1_	S	G_2_/M
Control	75.9 ± 5.8	10.4 ± 0.5	13.6 ± 1.2	91.6 ± 2.3	2.7 ± 0.2	5.8 ± 1.8	81.1 ± 3.9	7.4 ± 0.7	11.6 ± 1.1
Cisplatin (3 *μ*M)	45.9 ± 5.7	39.2 ± 1.6	14.8 ± 4	71.5 ± 2	18.2 ± 1.5	10.2 ± 1	24.1 ± 1.2	63.9 ± 2.4	11.9 ± 0.9
Sunitinib malate (1 *μ*M)	77 ± 4.5	11.7 ± 0.8	11.2 ± 1.7	93.7 ± 1.1	2.2 ± 0.1	4 ± 0.1	72.1 ± 2.4	11.6 ± 0.6	16.3 ± 1.6
Sunitinib malate (2 *μ*M)	78.7 ± 3.8	10.9 ± 1.4	10.1 ± 1.3	93.7 ± 2.2	2.4 ± 0.2	4.2 ± 0.5	71.6 ± 0.8	13.2 ± 0.2	15.1 ± 0.5
Sunitinib malate (4 *μ*M)	78.2 ± 3.3	12.5 ± 0.9	9.2 ± 1.2	93.9 ± 3.5	2.5 ± 0.2	3.5 ± 0.1	69.7 ± 2.7	14.5 ± 0.1	15.8 ± 1.1
Cisplatin (3 *μ*M) + Sunitinib malate (1 *μ*M)	52.4 ± 0.4	35.6 ± 0.7	11.9 ± 0.4	72 ± 3.2	18.6 ± 1.4	9.4 ± 1.5	32.1 ± 0.3	61.5 ± 3.4	6.4 ± 0.7
Cisplatin (3 *μ*M) + Sunitinib malate (2 *μ*M)	56.4 ± 2.9	34.4 ± 3.2	9.1 ± 0.5	74.6 ± 3.6	17.1 ± 1.3	8.3 ± 0.8	39.7 ± 3.2	54.2 ± 6.1	6.1 ± 0.4
Cisplatin (3 *μ*M) + Sunitinib malate (4 *μ*M)	56.8 ± 1.8	34.3 ± 1.2	8.8 ± 0.3	74.9 ± 2.7	12.2 ± 1	7.8 ± 2	38.8 ± 5.1	54.9 ± 2.9	6.2 ± 0.7

SD: standard deviation.

**Table 3:** Sub-G_0_/G_1_-fraction of HT1376, T24, and 5637 urinary bladder-cancer cell lines, after treatment with cisplatin and sunitinib malate, in isolation or combined. Sub-G_0_/G_1_ values are mean ± SD of the three independent experiments.

	Sub-G_0_/G_1_-fraction		
	HT1376	T24	5637
Control	13.1 ± 4.8	13.5 ± 4	8.6 ± 4.1
Cisplatin (3 *μ*M)	13.7 ± 1.1	22.6 ± 3.3	26.4 ± 2.1
Sunitinib malate (1 *μ*M)	35.9 ± 7.4	12.9 ± 1.2	46.4 ± 5.2
Sunitinib malate (2 *μ*M)	36.5 ± 3.2	13.6 ± 2.4	44.2 ± 1.8
Sunitinib malate (4 *μ*M)	44.2 ± 5.4	18.2 ± 3.4	35.3 ± 3.6
Cisplatin (3 *μ*M) + sunitinib malate (1 *μ*M)	24.5 ± 0.8	22.8 ± 6	33.4 ± 4.4
Cisplatin (3 *μ*M) + sunitinib malate (2 *μ*M)	47.4 ± 6.6	28.8 ± 5.2	34.5 ± 3.6
Cisplatin (3 *μ*M) + sunitinib malate (4 *μ*M)	49.3 ± 2.9	31.2 ± 5.7	34 ± 2.2

SD: standard deviation.
